# Trunk muscle activity during pressure feedback monitoring among individuals with and without chronic low Back pain

**DOI:** 10.1186/s12891-020-03565-y

**Published:** 2020-08-22

**Authors:** Xin Li, Wai Leung Ambrose Lo, Song-wei Lu, Howe Liu, Ke-yu Lin, Jian-yang Lai, Le Li, Chu-huai Wang

**Affiliations:** 1 Department of Rehabilitation Medicine, The First Affiliated Hospital, Sun Yat-sen University, Guangzhou, 510080 China; 2 Department of Physical Therapy, University of North Texas Health Science Center, Fort Worth, TX 76101 USA

**Keywords:** Low back pain, Multifidus, Pressure biofeedback unit, Transverse abdominals

## Abstract

**Background:**

Pressure biofeedback unit (PBU) is a widely used non-invasive device to assist core muscle training by providing pressure feedback. The aim this study was to compare the muscle activities of transverse abdominis (TA) and multifidus (MF) at different target pressures (50, 60 and 70 mmHg) of PBU between individuals with and without cLBP.

**Methods:**

Twenty-two patients with chronic LBP (cLBP) and 24 age matched healthy individuals were recruited. Electromyography (EMG) signals were recorded from the TA and MF muscles while the TA and MF were contracted to achieve PBU pressure value of 50, 60 and 70 mmHg in random order. The average EMG amplitude (AEMG) of 3 replicate trials was used in the analysis after normalization to %MVIC. %MVIC is defined as the mean of the three AEMG divided by the AEMG of MVIC. Two-way ANOVA was performed to assess the effects of groups (healthy and cLBP) and the three different target pressures of PBU. Independent sample *t*-test was conducted to compare between the two groups. Spearman’s correlation analysis was performed in the cLBP group to determine potential correlations between EMG activity, NPRS and ODI.

**Results:**

The %MVIC of the TA and MF in the cLBP group were higher than the control group at each pressure value (*P*<0.05). During maximal voluntary isometric contraction (MVIC) of TA and MF, compared with healthy groups, cLBP subjects showed a decrease (TA mean = 47.61 *μV*; MF mean = 42.40 *μV*) in EMG amplitudes (*P* ≤ 0.001). The MVIC of MF was negatively correlated with Numerical Pain Rating Scale (*r* = − 0.48, *P* = 0.024) and Oswestry Disability Index (*r* = − 0.59, *P* = 0.004).

**Conclusions:**

We measured the trunk muscles activities at different PBU pressure values, which allows the individual to estimate trunk muscle contraction via PBU. Clinicians may be able to confer the data obtained through EMG recordings to adjust the exercise intensity of PBU training accordingly.

## Background

Low back pain (LBP) is a major contributor to global burden of disability [1]. Animal experiment and mathematical model of the spine indicated that sequential injuries and deep muscles weakness resulted in spinal instability [2], which is defined as a significant decrease in the capacity of the spinal stabilizing system to maintain the intervertebral neutral zone within physiological limits [3]. Clinical guidelines suggested that spinal stabilization exercises (SSEs) may be as effective as other physiotherapy treatments in reducing disability and pain [4, 5]. The principle of SSEs is to train the co-contraction pattern of the deep local trunk muscles of the transversus abdominis (TA) and the multifidus muscles (MF). Despite the popularity to train deep muscles co-contraction to improve spinal stability and pain reduction, this theory remains controversial as fundamental evidence is lacking [6]. Early literature reported that the intrinsic muscle stiffness was not sufficient to stabilize the spine without neuromuscular reflex response (such as under a complex muscle synergy scenario) [6], which is accounted for 42% of the total stabilizing trunk stiffness [7]. In addition, some scholars suggested that it was the intra-abdominal pressurization, rather than the force activation of the selected abdominal muscles, that contribute to additional lumbar stability [8]. Ultrasonography study also indicated that delay onset of the abdominal muscles was not always present in people with non-specific LBP [9]. Thus, there also is a lack of uniformity regarding the meaning of “spinal stabilization” and what therapeutic exercises may be most effective [6–8].

The quantitative measurement tool of MF and TA plays a crucial role in assessing muscle activation pattern and clinical effectiveness of SSEs. The gold-standard to measure the activity of deep local trunk muscles is by fine-wire electromyography [7, 8]. However, factors such as pain, discomfort and risk of infection limit its clinical application as routine outcome measure. Indirect measurements of MF and TA muscles functions rely on electromyography (EMG) and ultrasound imaging. Surface EMG also has the limitation of cross-talk with other muscles that are in close proximity [8]. High cost and inconvenient hinders the common use of ultrasound imaging in clinical practice and the assessment of ultrasound is often limited by the position of the subject [10]. In clinical and research setting, PBU is a non-invasive, low-cost and convenient to use device that has been used to monitor the change of pressure as a mean to estimate the muscle activation of the MF and TA muscles during specific maneuverer [10, 11]. An inability to maintain the required pressure while performing the posture is reflective of an inability to maintain abdominal muscle contraction, resulting in uncontrolled movement and instability of the lumbar spine [12]. The validity of such approach was to investigate in early literature which reported moderate correlation between changes and PBU pressure and EMG activities [13]. The inter- and intra-examiner reproducibility of PBU in measuring TA muscle activity in people with cLBP [14] and healthy [11] individuals was reported to be excellent.

Previous studies suggested that the pretrial pressure of 70 mmHg was suitable for prone position [10, 14] and 40 mmHg was suitable for hook-lying supine [11], side-lying [15] and upright positions [11]. However, few studies were conducted in seated position. In Australia, 70% of adults sit for more than 8 h per day [16], and the time is likely to extend due to the increasing use of social media [17]. Prolonged sitting seems to be unavoidable in our modern daily life, work and study. Training the TA and MF muscles with PBU in the correct seated position could increase lumbar stability, which might be one of the measures to reduce the occurrence of LBP [11, 18]. To improve the clinical application of PBU and facilitate interpretation of activation of deep local trunk muscles, further investigations are needed to establish the response between activity of trunk muscles and different pressures of PBU in seated position.

This study aimed to answer the research question of whether changes in PBU pressure measured during MF and TA contraction in a seated position may induce corresponding changes in EMG muscle activities. The study also investigated if people with cLBP have different muscle activation pattern of TA and MF in a seated position, and whether changes in muscle activation pattern is associated with pain. We hypothesized that muscle activation patterns were significantly different between people with cLBP and heathy individuals, and the differences in activation pattern were associated with pain and disability.

## Method

### Subjects

Subjects were recruited from the local rehabilitation ward and outpatient department of the hospital. The inclusion criteria of cLBP subjects were as follows: 1) experienced pain in the low back region with or without accompanying buttock pain over the past 3 months, and of sufficient intensity to have limited activities of daily living [14]; 2) pain score range between 3 and 7 on the Numerical Pain Rating Scale (NPRS) [18]; and 3) able to perform the experiment procedure without symptom aggravation. This was to minimize the variability in the level of pain during testing which may increase the variability of the data. Exclusion criteria were: 1) existence of respiratory, orthopaedic, circulatory or neurological conditions; 2) previous surgery to the abdomen or lower back; 3) female subjects who were pregnant or suffered from dysmenorrhea; 4) epilepsy or had family history of epilepsy. The exclusion criteria of NPRS higher than 7 was due to published literature indicated patients who have NPRS 7 or above were not able to perform maximum contraction [14, 18]. As maximum contraction was necessary in this study to obtain the MVIC EMG data, this exclusion criteria was adopted. Age matched healthy individuals with no existing LBP and no LBP in the past year were recruited as control.

### Ethics

The study protocol was reviewed and approved by the Human Subjects Ethics Sub-committee of the First Affiliated Hospital, Sun Yat-sen University (Approval number: [2017] C-034). All subjects gave written informed consent. The study was conducted in accordance to the Declaration of Helsinki.

### Instruments

The Pressure Biofeedback Unit (PBU) (Chattanooga Group Inc., LLC Vista, California, USA) employed in this study is made up of a three-chamber air-filled pressure cell, a catheter and a sphygmomanometer gauge. The pressure cell of the PBU was made from latex-free rubber material, and the unfolded dimension of the cell was 16.7 × 24.0 cm. The sphygmomanometer was calibrated to 2 mmHg interval and has a range between 0 to 200 mmHg. Movement or change in position causes volume change in the pressure cells that is displayed on the gauge. Prior to data recording, the pressure cell was first inflated to a pressure of 40 mmHg (orange band). The valve was then closed to stop air leakage [11]. To ensure accuracy of the PBU measurements, the device was pretested by loading a static weight of 4 kg for 24 h. The PBU was only considered adequate if the device lost no more than 0.5 mmHg during the 24-h period [19].

Muscle activity was assessed by the gold standard surface EMG (UMI-SE-I sEMG system, Shaoxing United Medical Instruments Co., LTD, China). EMG was adopted to study the activity of TrA due to it being regarded as the gold standard to measure muscle activities, despite its limitation of cross-talk with muscle fibers that are in close proximity. Ultrasonography was not suitable for the present study as the ultrasound probe could not be placed on the location that was required to scan the TrA in a seated position. EMG signals with common mode rejection ratio of 110db, bandwidth of 15–1000 Hz and resolution of 0.1 μV. Sampling frequency was set at 3000 Hz and stored in a computer for offline analysis [18]. To reduce skin impedance, hair was removed from the measurement sites and the skin was deterged with alcohol before electrode placement. Disposable Ag/AgCl surface electrodes were attached to the concerned muscle. The maximum space between the recording electrodes was 2 cm. The locations of the EMG electrodes were determined in accordance to EMG placement guidelines [20] and published studies [6, 7, 14]. Electrodes to measure TA activity were placed at the center position that was 2 cm cephalic to the pubic bone, just lateral to the midline, and parallel to the superior pubic ramus along either side of the course of the underlying muscle fibers [14, 20]. For MF, electrodes were placed at the level of the L5 spinous process along the line joining the posterosuperior iliac spine (PSIS) and L1–L2 vertebral interspace [18, 20]. To obtain the MVIC data of the TA, subjects were in sit-up styled movements with both legs fixated on the floor, using a belt or manually fixed. The spine then flexed to 30° and maintained the position for 5 s to record MVIC [20]. To obtain the MVIC data of MF, the subjects laid in prone position on a standard plinth with the upper limbs positioned overhead. Subjects then lifted their head and upper extremities with maximum effort and maintained the position for 5 s to record MVIC [18, 20]. The maximal voluntary isometric contraction (MVIC) was measured to determine the level of voluntary contractions during testing. MVIC was repeated 3 times and the highest value was selected for analysis [11, 20]. The average EMG amplitude (AEMG) at each pressure level was obtained from 3 repeated trials. AEMG was used in the data analysis after normalization to %MVIC [11, 20], which was calculated for the selected muscles using the formula: % MVIC= $\frac{AEMG}{MVIC}\times 100\%$. The slope of the %MVIC-pressure relation was calculated at 70 mmHg and 50 mmHg pressure and compared between the two groups. The rationale to choose AEMG and %MVIC as variables was that raw EMG readings were not comparable between individuals. By normalizing the MVC, the raw EMG reading was rescaled to a percentage of a reference value that was standardized across all individuals within the study. The standardized MVC values enabled quantitative comparisons of the EMG readings between individuals.

### Experimental procedures

Demographics and background clinical information were first collected. Participants were asked to score the pain intensity over the last 24 h on the NPRS. The scale range between 0 and 10, with 0 being no pain and 10 being severe pain [21]. Pain related disability was assessed by the Chinese version of the Oswestry Disability Index (ODI) [22]. The total score of ODI ranges from 0 to 100, with 0 being no disability and 100 being maximum disability [22].

All subjects then received information about the anatomy, biomechanics and functions of the TA and MF muscles. All subjects were asked to fast for at least 2 h, avoid performing any type of abdominal exercises, and empty their bladders prior to data collection [10]. The procedures of PBU tests were described in Table 1. They were performed in random order by selecting a single card with three cards marked with 1 (50 mmHg), 2 (60 mmHg), or 3 (70 mmHg). The examiner provided verbal instructions throughout the test. Subjects were asked to maintain within ±2 mmHg from the target pressure [11]. The accuracy of maintaining the required PBU pressure was confirmed by visual inspection of the gauge.

**Table 1:** Methods for positions of interest muscles and EMG signals

	Description of the methods	Positions
Transversus Abdominis (TA)	Subjects were seated upright against the wall with one hand holding the sphygmomanometer gauge and both feet rested on the floor. The pressure cell was placed behind the lumbar spine. Subjects slowly pulled their lower abdomen and navel towards the back to contract TA without changing spinal position change [11, 14]. The PBU pressure of 50 mmHg, 60mmHg and 70mmHg were selected in random order.	
Multifidus (MF)	Subjects were seated uprightly against the wall with one hand holding the sphygmomanometer gauge and both feet rested on the floor. The pressure cell was placed behind the medial edges of the shoulder blades. Subjects slowly extended the lumbar spine to contract MF [18, 20], until the PBU pressure reached 50 mmHg, 60mmHg or 70mmHg.	

All subjects in the pain-free group selected the muscles on the right side. Subjects in the cLBP group chose the more painful side as target muscles. Three trials were conducted for each target pressure and the averaged values were used for analysis. A resting interval of 30 s was provided between each trial to minimize fatigue. Participants were allowed to stand up and move during the interval.

### Statistical analysis

Statistical analysis was conducted in the statistical Package for the Social Sciences version 25.0 software (SPSS Inc., Chicago, IL, USA). The EMG data at different PBU pressures were expressed as mean and standard deviation. The Shapiro–Wilk test was used to test the normality distribution of the data. Wilcoxon’s signed-rank test was used for non-normal distribution parameters. Independent sample t-test was conducted to compare subject characteristics. Two-way ANOVA was performed to assess the effects of groups (Healthy and cLBP) and the three different target pressures (50, 60 and 70 mmHg) of PBU. If the main effect of the pressure was significant, a post hoc test was performed using the Bonferroni correction. If the main effect of the groups was significant, independent sample *t*-test was conducted to compare between two groups. Spearman’s correlation analysis was performed in the cLBP group to determine the correlations between EMG activity, NPRS and ODI. A value of *P* < 0.05 was considered to be statistically significant.

## Results

Twenty-two right-handed individuals were recruited in cLBP group and 24 age matched healthy individuals were recruited in the control group. The sample characteristics of both cohorts are presented in Table 2. There was no group difference for gender, age, height, weight, BMI and educational level. Two cLBP individuals were unable to maintain 70 mmHg of TA and were excluded from the analysis. Twenty cLBP participants were included in the analyses of TA at the pressure value of 70 mmHg (Table 3).

**Table 2:** Characteristics of the sample cohorts (mean (SD))

Demographics	cLBP (*n* = 22)	Pain-free controls (*n* = 24)	*P*-value
Gender (M: F)	6:16	5:19	0.613
Age (years)	28.27 (8.15)	25.17 (4.00)	0.103
Height (cm)	163.55 (9.22)	163.88 (7.24)	0.893
Weight (kg)	57.86 (7.47)	54.92 (6.56)	0.277
BMI (kg/m^2^)	21.59 (1.78)	20.25 (1.79)	0.059
Education level (years)	15.36 (2.52)	15.58 (0.78)	0.686
Side of pain (L: R)	9:13	**–**	**–**
Pain intensity (NPRS)	4.73 (1.45)	**–**	**–**
Pain duration (years)	3.65 (5.33)	**–**	**–**
ODI (%)	25.64 (11.85)	**–**	**–**

*Key: cLBP* chronic Low Back Pain, *BMI* body mass index, *ODI* Oswestry disability index, *L* left, *R* right, *NPRS* numerical pain rating scale, *SD* standard deviation

**Table 3:** At each target pressure value, mean and standard deviation (SD) of AEMG (*μV*) and MVIC for TA and MF muscles

Interest muscles at target pressure value	cLBP (n = 22)	Pain-free controls (n = 24)	*P*
Transversus Abdominis (TA)			
MVIC	49.44 (22.98)	97.05 (55.09)	<0.001
50 mmHg	13.75 (12.41)	11.60 (8.88)	0.792
60 mmHg	18.85 (13.93)	16.16 (13.66)	0.468
70 mmHg (*n* = 20/22)	23.38 (16.01)	19.58 (14.84)	0.437
Multifidus (MF)			
MVIC	61.38 (37.21)	103.78 (42.29)	0.001
50 mmHg	11.55 (7.91)	11.19 (7.59)	0.886
60 mmHg	17.94 (9.63)	18.16 (12.3)	0.843
70 mmHg	26.60 (13.36)	27.86 (18.23)	0.912

*Key: cLBP* chronic Low Back Pain, *MF* multifidus, *MVIC* maximal voluntary isometric contraction, *TA* transversus abdominis

### %MVIC at different pressure values of PBU training

**Fig. 1:**
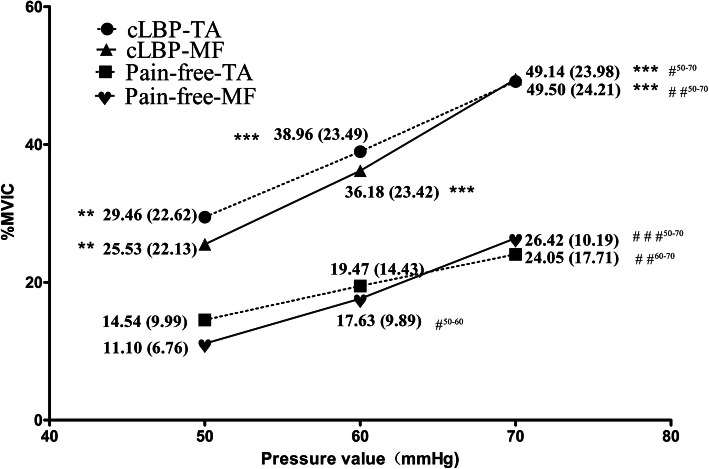
The association between deep local trunk muscles (TA or MF) and different target pressures of PBU at seating position, and the different for the local trunk muscles (TA or MF) between individuals with and without cLBP. cLBP, chronic Low Back Pain; EMG, electromyography; MF, Multifidus; MVIC, maximal voluntary isometric contraction; TA, Transversus Abdominis; * indicates the difference between cLBP group and the pain-free group; * *p* < 0.05; **p < 0.01; *** *p* < 0.001; # indicates the difference between different pressures; # p < 0.05; # #p < 0.01; # # # p < 0.001

Figure 1 shows the response between TA and MF muscle activities at different target pressures of PBU, and the differences in TA and MF between individuals with and without cLBP. For TA, the results of two-way ANOVA tests indicated no statistically significant difference among the two groups and different pressures (*F*_interaction-effect_ = 0.825, *P* = 0.440), the main effect of the pressure was not significant either (*F*_pressure-main_ = 0.198, *P* = 0.821), and the groups effect was significant (*F*_group-main_ = 4.500, *P* = 0.036). Similar results were found in MF (*F*_interaction-effect_ = 0.713, *P* = 0.492; *F*_pressure-main_ = 0.071, *P* = 0.932; *F*_group-main_ = 7.569, *P* = 0.007). The %MVIC of the TA and MF in the cLBP group were statistically higher than the control group at every pressure value (*P*<0.05). In both groups, the %MVIC of TA were more active than the MF at 50 mmHg and 60 mmHg. At 70 mmHg, the cLBP group had almost equal activity in both muscles, while the MF %MVIC of the healthy group had higher activity than TA %MVIC.

Both the TA (*F* = 3.721, *P* = 0.03) and the MF (*F* = 5.86, *P* = 0.005) %MVIC of cLBP group showed significant differences between 50 mmHg and 70 mmHg. In the pain-free group, there were statistically significant differences in MF %MVIC under each of the three pressure (*F* = 17.202, *P*<0.001), while there was no difference in TA %MVIC under each pressure (*F* = 1.131, *P* = 0.329).

### Average EMG amplitudes (AEMG)

During MVIC of TA and MF, cLBP group were significantly less than the healthy group (*P* ≤ 0.001). No difference was observed in the AEMG at any target pressure value (*P* > 0.05). Table 3 illustrates the mean and standard deviation of AEMG and MVIC for the TA and the MF at each target pressure.

### Correlation between EMG activity, NPRS and ODI

**Fig. 2:**
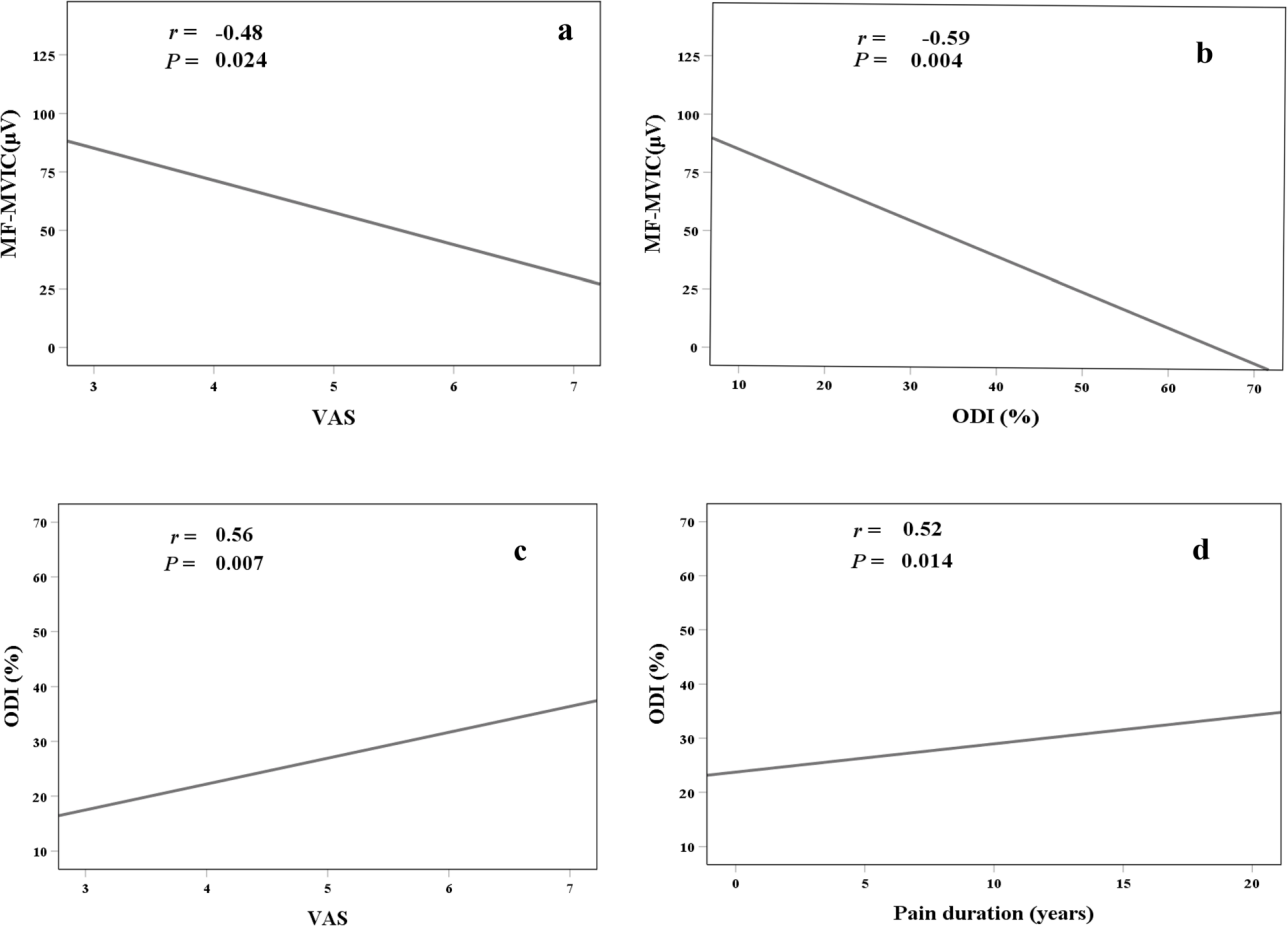
Correlation between EMG activity and NPRS, ODI. cLBP, chronic Low Back Pain; MF, Multifidus; MVIC, maximal voluntary isometric contraction; ODI, Oswestry Disability Index; TA, Transversus Abdominis; NPRS, Numerical Pain Rating Scale

MF MVIC was negatively correlated with NPRS (*r* = − 0.48, *P* = 0.024) (Fig. 2a) and ODI (*r* = − 0.59, *P* = 0.004) (Fig. 2b). No significant correlation between TA MVIC and NPRS (*r* = − 0.12, *P* = 0.591), or between TA MVIC and ODI (*r* = − 0.26, *P* = 0.250) were observed. Both of the MF MVIC and TA MVIC were not significantly correlated with pain duration. Other EMG activities (%MVIC and AEMG of TA, MF at any target pressure) were not significantly correlated with NPRS, ODI and pain duration. ODI was positively correlated with NPRS (*r* = 0.56, *P* = 0.007) (Fig. 2c) and pain duration (*r* = 0.52, *P* = 0.014) (Fig. 2d). Figure 2 illustrates the correlation between EMG activity, NPRS and ODI.

## Discussion

The present study observed that PBU measurement during MF and TA contraction in a seated position induced corresponding changes in EMG muscle activities. Muscle activities of the TA and MF during MVIC were lower in the cLBP group than the control group. This indicated that people with cLBP were unable to generate as much force as healthy individuals. However, muscle activities were significantly higher at each pressure value in the cLBP group than the control group. This indicated a stronger contraction was required to maintain spinal stability in people with cLBP. The MVIC of MF was negatively correlated with NPRS and ODI. TA MVIC was not correlated with NPRS or ODI.

### MF and TA muscle activities in healthy controls vs cLBP subjects

Our results indicated that the %MVIC of the TA and MF in the cLBP group were statistically higher than the control group at every pressure value. For MF, finding of our present study was consistent with previous research published by Ansari et al. [23] which explained the high %MVIC of MF. They suggested that muscle pain could be accompanied by hyperactivity in the back muscles during dynamic conditions, which was known as the pain adaptation model [23]. The significantly reduced MVIC of the MF muscle in cLBP group might also support this phenomenon. However, the mechanism of MVIC reduction is not totally understood, but may be related to pain inhibition which limits the ability to perform maximum muscle contraction. People with cLBP have a greater sensitivity to pain [24]. Thus, we speculate that cLBP alters spontaneous neuronal activity resulting in changes in EMG activity [18]. Additionally, atrophic changes of MF had been confirmed in around 77–80% of LBP cases, especially at the L5–S1 level [25] (the EMG site of MF in our study), which might contribute to lower MVIC in the cLBP group.

TA is an important deep muscle that plays a key role in the dynamic control of the lumbar spine [6, 18]. The present study observed lower TA EMG amplitudes during MVIC in the LBP group than the healthy group. Hodge et al. reported a delay in TA muscle contraction relative to the agonist muscle that moved the limbs in people with LBP [26]. Gildea et al. [27] reported that under contraction status, the thickness of TA was higher in female dancers with cLBP than those without pain. These data support our finding of low TA muscle activity during MVIC.

### Differences in muscle activation pattern

Our results showed that the TA %MVIC was more active than MF %MVIC at 50 mmHg and 60 mmHg in both groups. These findings are consistent with published studies that investigated the relationship between TA and MF in patients with cLBP [28]. The study reported that patients who had adequate contraction of multifidus were of 4.5 times likely to be able to contract TA. At the PBU pressure of 70 mmHg, the cLBP group demonstrated almost equal muscle activity in both TA and MF muscles, whereas in the healthy group the %MVIC of MF had more activity than TA. The potential reason may be related to the fatigue of the multifidus muscle. According to published literature [25, 29], sensorial factors influence the recruitment of TA and contributed to MF fatigue. The study by Ramos et al. [30] utilized surface EMG to assess fatigue of MF and PBU to detect activity of TA in patients with LBP. They reported that patients with LBP had difficulties to depress the abdominal wall at the PBU pressure 70 mmHg and higher. MF fatigue was also observed. Another possible reason was that cLBP patients had reduced flexibility and mobility in the frontal, transverse, and sagittal planes of motion [31]. When TA was contracted at 70 mmHg, there was limited space in the anatomical position [31] that the low back required to complete the motion.

### Correlation between muscle activities, pain and disability

The present study observed a negative correlation between MVIC of MF and NPRS and ODI which was consistent with previous studies [18]. There was no correlation between the MVIC of TA and NPRS or ODI. The potential reason was that TA and MF have different roles in maintaining lumbar stability due to their different anatomical structures, different muscle fiber size, different motor unit control properties [24, 25]. Previous studies suggested that the TA was mainly involved in lumbar stability by contractile increase of abdominal pressure [10, 14], and MF directly maintain lumbar stability through the thoracolumbar fascia [5, 24, 32]. Therefore, compared with the TA, MF might be more correlated with NPRS and ODI. Moreover, TA and MF had different neuromuscular and proprioceptive systems, along with varied changes in biomechanical alignment of the spine and developed different models of pain adaptation [33]. However, speculation on the connection between neuromuscular control mechanisms and pain was difficult as little is known about the underlying relationship between brain network and the TA and MF muscle activity [28].

### Limitations

First, the application of PBU to indirectly estimate muscle activity is a limitation. The measurement of pressure simply indicates whether an individual is able to maintain a constant pressure while performing a particular activity. It is inconclusive if the measurement properties of PBU for the assessment of TA activity is adequate. Therefore, PBU measurement should not be used solely as a mean to estimate muscle activity. Surface EMG measured muscle activities has the limitation of cross-talk from muscles that are in close proximity. Although ultrasonography does not have such limitation and may be more appropriate to study TA activity, however, it was not technical feasible to perform ultrasound scan of the TA in a seated position. The reliability and validity of the PBU and EMG in measuring MF muscle activity in patients with cLBP were not established. Further studies are needed to assess the reliability and validity of this method for evaluating MF. Second, cLBP may interfere with the person’s ability to perform maximum muscle contraction. Individuals may therefore not able to perform their “maximum” ability during MVIC due to pain aggravation. The assessor provided detail instructions and verbal encouragements in the present study as an attempt to minimize the impact. The uneven distribution of gender in the sample population might be also a limitation.

## Conclusions

We measured the trunk muscles activities at different PBU pressure values, which allows the individual to estimate trunk muscle contraction via PBU. Clinicians may be able to confer the data obtained through EMG recordings to adjust the exercise intensity of PBU training accordingly.

## Data Availability

The dataset supporting the conclusions of this article is available from the authors upon request.

## Abbreviations

BMI: Body mass index
cLBP: chronic low back pain
EMG: Electromyography
ICC: Intraclass correlation coefficient
MF: Multifidus
MVIC: Maximal voluntary isometric contraction
ODI: Oswestry disability index
PBU: Pressure biofeedback unit
PSIS: Posterosuperior iliac spine
SD: Standard deviation
SSEs: Segmental spinal stabilization exercises
TA: Transversus abdominis
NPRS: Numerical pain rating scale
